# A Novel Ultrasound Technique for Detection of Osteochondral Defects in the Ankle Joint: A Parametric and Feasibility Study

**DOI:** 10.3390/s150100148

**Published:** 2014-12-24

**Authors:** Nazli Sarkalkan, Arjo J. Loeve, Koen W. A. van Dongen, Gabrielle J. M. Tuijthof, Amir A. Zadpoor

**Affiliations:** 1 Department of Biomechanical Engineering, Faculty of Mechanical Engineering, Delft University of Technology (TU Delft), Mekelweg 2, Delft 2628 CD, The Netherlands; E-Mails: a.j.loeve@tudelft.nl (A.J.L.); g.j.m.tuijthof@tudelft.nl (G.J.M.T.); a.a.zadpoor@tudelft.nl (A.A.Z.); 2 Department of Imaging Physics, Faculty of Applied Sciences, Delft University of Technology (TU Delft), Lorentzweg 1, Delft 2628 CJ, The Netherlands; E-Mail: k.w.a.vandongen@tudelft.nl; 3 Department of Orthopedic Surgery, Academic Medical Centre (AMC), Meibergdreef 9, Amsterdam 1105 AZ, The Netherlands

**Keywords:** diagnosis, (osteo)chondral defect, ankle joint, joint space, ultrasound propagation, 2D finite-difference time-domain model

## Abstract

(Osteo)chondral defects (OCDs) in the ankle are currently diagnosed with modalities that are not convenient to use in long-term follow-ups. Ultrasound (US) imaging, which is a cost-effective and non-invasive alternative, has limited ability to discriminate OCDs. We aim to develop a new diagnostic technique based on US wave propagation through the ankle joint. The presence of OCDs is identified when a US signal deviates from a reference signal associated with the healthy joint. The feasibility of the proposed technique is studied using experimentally-validated 2D finite-difference time-domain models of the ankle joint. The normalized maximum cross correlation of experiments and simulation was 0.97. Effects of variables relevant to the ankle joint, US transducers and OCDs were evaluated. Variations in joint space width and transducer orientation made noticeable alterations to the reference signal: normalized root mean square error ranged from 6.29% to 65.25% and from 19.59% to 8064.2%, respectively. The results suggest that the new technique could be used for detection of OCDs, if the effects of other parameters (*i.e.*, parameters related to the ankle joint and US transducers) can be reduced.

## 1. Introduction

The ankle joint is one of the common locations for (osteo)chondral defects (OCDs) representing disruption of articular cartilage together with or without disintegration of the subchondral bone [1–3]. These defects can occur due to acute trauma, repetitive micro-trauma, and torsional joint loading during sport [3–5] and domestic activities (e.g., a fall from stairs) [6]. If OCDs are left untreated, there is a potential risk of developing early osteoarthritis (OA) [1,7]. As OA is a disease decreasing individuals' quality of life and imposes a huge socioeconomic burden on society [8], early detection and treatment of OCD is highly important.

The techniques currently used for detection of OCDs are computed tomography (CT), magnetic resonance imaging (MRI), and arthroscopy [3,9–11]. Although these techniques are sensitive in detection of defects [11], they are not favorable to be used in longitudinal follow-up of individuals due to ionizing radiation (e.g., in CT), long acquisition time (e.g., in MRI), and invasiveness (e.g., arthroscopy) [10] as well as the costs involved. Ultrasound (US) imaging, which is known to be non-invasive, fast, and cost-effective [12], is not extensively used in diagnosis of OCDs. That is primarily because US imaging is not capable of successfully imaging most cartilage defects [13] because of the limited ability of US waves to penetrate through bone [14].

Keeping cost-effectiveness and non-invasiveness of US in mind, an acoustic wave method which does not require penetration of sound waves through bone might be a good alternative to the currently-used diagnostic techniques. We propose a new diagnostic technique, which is based on the propagation of US waves through the entire joint space of the ankle and on definition of changes in acoustic wave response properties ensued as a result of defect. Specification of acoustic parameters that can describe the entire joint space morphology and be robust to individual variations will be necessary for successful identification of OCDs. However, determination of optimal parameters is a major challenge, as little is currently known about the US propagation in the joint space of the ankle and effects of many parameters on the acoustic wave characteristics.

The aim of this work was to study US propagation in the joint space of the ankle and to determine the feasibility of the new concept for detection of defects. To reduce the complexity of the problem, simplified 2D finite-difference time-domain (FDTD) models of ankle joint were developed and effects of dominant variables relevant to the ankle joint (*i.e.*, joint space width), US transducer (*i.e.*, translation and rotation of US transducer acting as transmitter) and defect (*i.e.*, width, depth and location) on acoustic wave response were evaluated. An experiment was performed to validate a FDTD model, which represents the healthy state of the joint. To the authors' knowledge, this is the first study to evaluate propagation of US in the joint space of the ankle and to determine the feasibility of such a concept for detection of OCDs.

## 2. Materials and Methods

**Figure 1.**
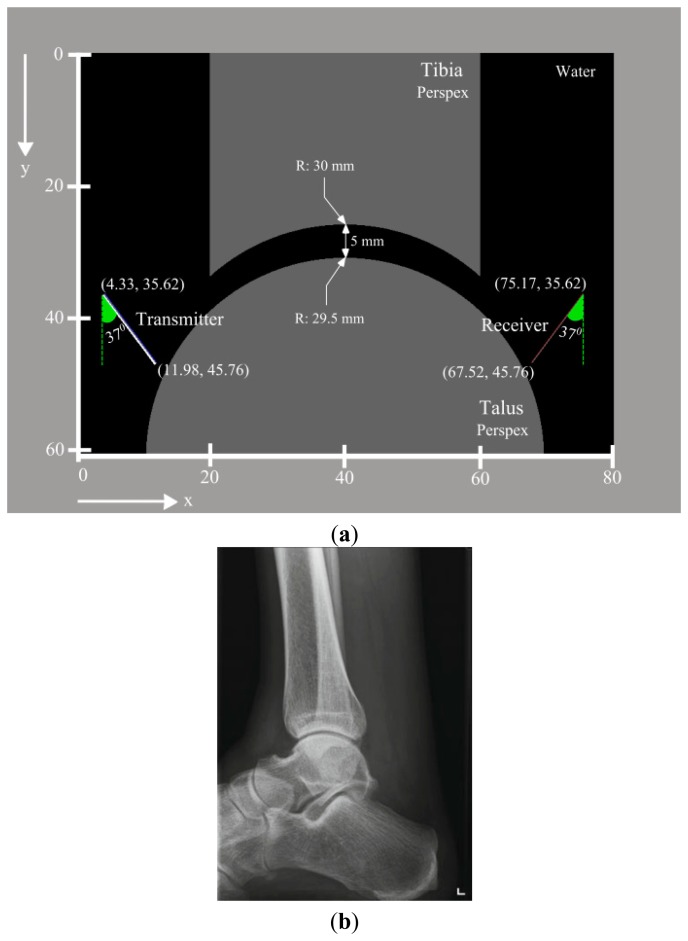
(**a**) Representation of the reference simulation model. Joint space width of the ankle is 5 mm. The radii of the tibial and talar arcs respectively are 30 mm and 29.5 mm. US probes are positioned in a nearly identical manner to the experiment; (**b**) Radiograph of a healthy ankle joint—Lateral view [15].

**Figure 2.**
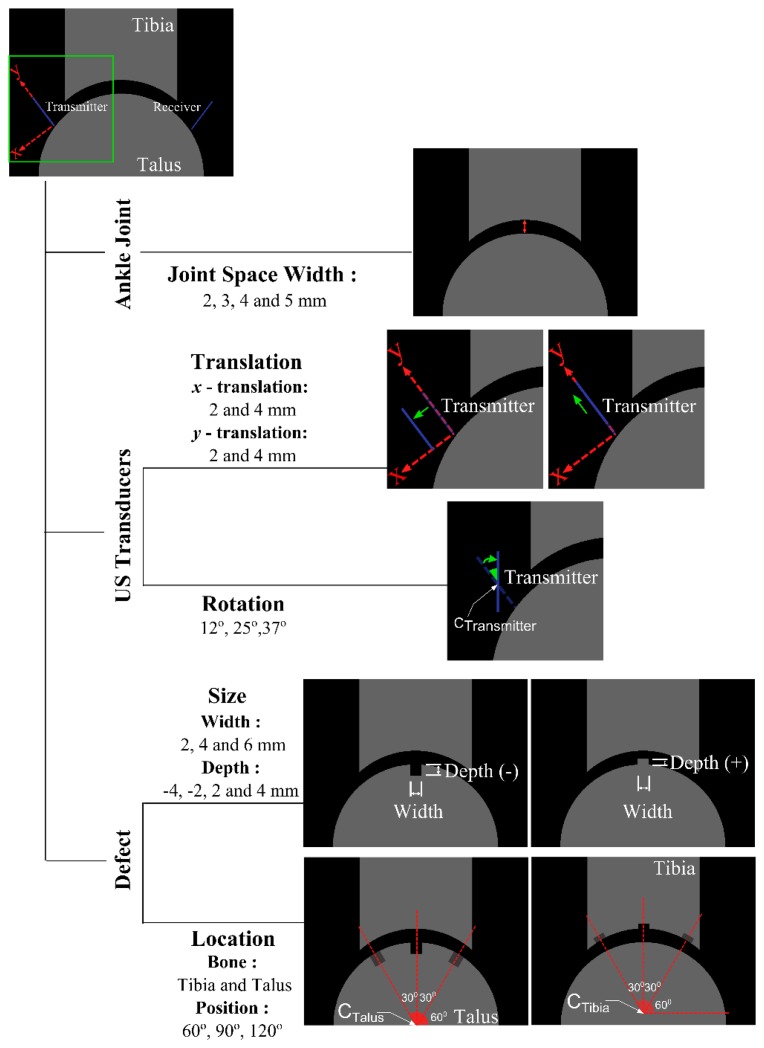
Representation of parameters related to the ankle joint (*i.e.*, joint space width), US transducers (*i.e.*, translation and rotation of US transducer acting as transmitter) and defect (*i.e.*, width, depth and location). Joint space width values are varied from 2 mm to 5 mm (*i.e.*, reference simulation model) in intervals of 1 mm. Translations of US transducer were ranged from 2 mm to 4 mm. The US transducer rotated 37° around its center implies that it is perpendicularly aligned to the talar surface, whereas the other two angles were arbitrarily chosen: 12°, and 25°.

Throughout this study, two series of simulations and one series of experiments were performed. The first set of simulations incorporates a reference model (Figure 1a), which mimics the healthy state of the joint and was validated with experimental measurements. The second series includes models generated to evaluate the effects of main variables (Figure 2) related to the ankle joint (*i.e.*, joint space width), US transducers (*i.e.*, translation and rotation of US transducer acting as transmitter), and defect (*i.e.*, width, depth and location) on the output signal of the reference model.

### 2.1. Simulations

All the simulations were performed using the US simulation software Wave2000 (Cyberlogic Inc., New York, NY, USA). To find an approximate solution to the 2D acoustic wave equation, the software uses the algorithm presented by Schechter *et al.* [16]. In brief, this algorithm assumes that a heterogeneous medium is composed of homogenous linear isotropic regions and imposes continuity of stresses and displacements across boundaries of four homogenous regions. Within each homogeneous grid element, an acoustic differential equation is solved and the displacement vector is computed at the intersection of four grid elements at each time step of the simulation:

(1) $$\rho \frac{\partial^{2}\text{w}}{\partial t^{2}}=\left|\mu +\eta \frac{\partial }{\partial t}\right|\nabla^{2}\text{w}+\left|\lambda +\mu +\phi \frac{\partial }{\partial t}+\frac{\eta }{3}\frac{\partial }{\partial t}\right|\nabla \left(\nabla \cdot \text{w}\right)$$

where **w** is a 2D vector whose components are the *x* and *y* components of displacement of the medium at location (x, y), ρ = material density [kg/m^3^], λ = first Lamé constant [N/m^2^], μ = second Lamé constant [N/m^2^], η = shear viscosity [N·s/m^2^], ϕ = bulk viscosity [N·s/m^2^], *t* = time [s], ∂ = the partial difference operator, ∇^2^ = the Laplace operator, and ∇ = the divergence operator.

#### 2.1.1. Reference Model

The first step in generation of the model was definition of the geometry. As the reference model would be used in assessment of US propagation in the joint space of a healthy ankle joint, a simplified geometry (Figure 1a) which is similar in size and shape (Figure 1b) to the ankle joint was considered. The study of Stagni *et al.* was consulted for determination of a realistic size for the tibia and the talus [17]. Additionally, a joint space width of 5 mm (Figure 1a) was used, considering the fact that the ankle joint space can be increased up to 5 mm upon distraction loading [18,19]. A geometry file representing the simplified ankle joint was prepared using Matlab R2013b (The MathWorks, Inc., Natick, MA, USA) and imported into Wave2000.

The second main step in preparation of the model was the definition of the material properties. Following White *et al.* [20], Perspex was chosen to prepare the experimental samples (*i.e.*, tibia and talus) due to its ease of milling. Plexiglas material properties (Table 1), which were derived from the material library embedded in the Wave2000, were assigned to the parts representing the tibia and the talus (Figure 1a). In addition, the simplified ankle joint was considered to be submerged in water, representing the synovial fluid. Material properties of water (Table 1) were defined using the material library of the Wave2000 and incorporated in the simulation model (Figure 1a). All four sides of the simulation model boundaries were taken as expanding to infinity.

Two non-focused 1 MHz US transducers (diameter: 12.7 mm) were positioned (Figure 1a) and oriented similarly to the transducers in the experimental setup that was used to validate the simulation result. A uniform apodization factor was used for both the transmitter and the receiver. Default settings of the Wave2000 for gain (*i.e.*, 0 dB), blanking (*i.e.*, 0 μs), and duration (*i.e.*, 0 μs) were used for the receiver. As the simulations were intended to represent the experimental setup as closely as possible, the source signal (*i.e.*, time function of US transducer acting as transmitter) was defined using the procedures presented in the section describing the experimental setup and was used for all numerical simulations.

To determine the grid size, the prepared model was run a total of 11 times while gradually decreasing the grid size of the model from 100 μm to 16.7 μm. Subsequently, simulations results were compared to each other and the similarity between two subsequent simulations was computed using normalized root mean square errors (NRMSE):

(2) $$\begin{array}{l} \text{NRMSE}=\frac{\sqrt{\frac{\overset{n}{\underset{i=1}{\text{∑}}}{\left(x_{\text{obs},i}-x_{\text{ref},i}\right)}^{2}}{n}}}{x_{\text{obs},\text{max}}-x_{\text{obs},\min}}\times 100\% \end{array}$$

where *x_obs,i_* is the observed value at time *i*, *x_ref,i_* = reference value at time *i*, *x_obs,max_* = maximum of the observed data, *x_obs,min_* = minimum of the observed data, and *n* = number of data points. Simulation results were assumed to have converged if the NRMSE was less than 1%. Based on the simulation results, a grid size of 20 μm was found to result in convergence and was used throughout the study. The time step of 6.5 ns was automatically calculated by the software. The total simulation time was 100 μs.

#### 2.1.2. Parametric Models

**Figure 3.**
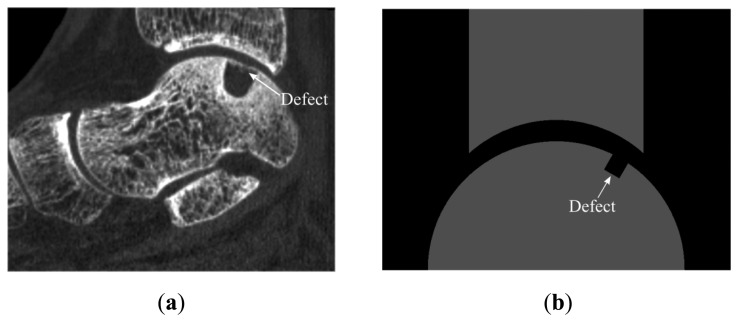
(**a**) Sagittal reconstructed image of CT scans of an ankle with OCD; (**b**) Representation of a defect in simulations.

Before going through the preparation of all the models that would be necessary to evaluate the effects of the parameters (e.g., variables related to the ankle joint) on the acoustic wave response, experiments were performed. The experimental results were used to investigate whether the reference simulation can represent reality sufficiently accurate. Reassured by a good agreement between the reference simulation results and experimental findings, all the parametric models were generated using the same procedures. Parameters expected to be most dominantly influencing the wave response were studied. These parameters were classified into three main categories: factors related to (I) the ankle joint geometry; (II) US transducers; and (III) defect (Figure 2). These categories consisted of one (*i.e.*, joint space width), two (*i.e.*, translation and rotation of US transducer acting as transmitter) and two (*i.e.*, size and location of the defect) categories, respectively. In addition, categories relevant to size and location of the defect and to translation of US transducer were all split into two subcategories (Figure 2). Only one parameter of the model was varied at each simulation to gain insight into the influence of each parameter on the output signals. With respect to the factors related to the ankle joint (Figure 2), the joint space width values were varied from 2 mm to 5 mm (*i.e.*, reference model) [18,21] in intervals of 1 mm. Moreover, OCDs (Figure 3a) were represented with a rectangular shape (Figure 3b) in the parametric models simulating parameters related to defect. The values used to describe depth and width of defects were respectively varied from 2 mm to 4 mm and 2 mm to 6 mm, thereby remaining within the range of values reported for OCDs [22]. In addition to the defects with negative depth values, positive defects (Figure 2), which may occur in the stage IV lesions (*i.e.*, detached and displaced osteochondral fragment) [23] were considered. Throughout the simulations, the size of temporal and spatial domain, the grid size, the boundary conditions, and the material properties were kept identical to the reference model.

### 2.2. Experiments

**Figure 4.**
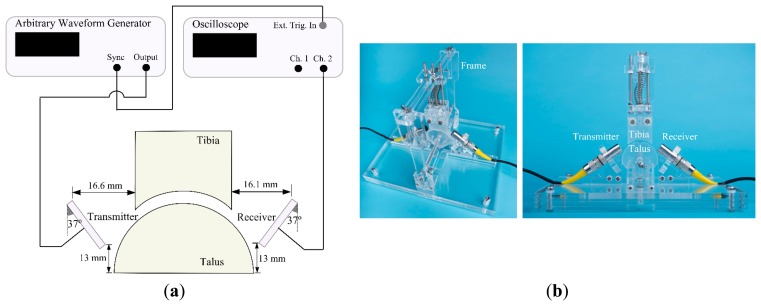
(**a**) Schematic presentation of the experimental setup; (**b**) Perspex frame designed to hold the tibia and the talus parts. Holders for transducers are included to fixate them at the desired positions.

The experimental setup used is depicted in Figure 4. It consists of two unfocused 1 MHz US transducers with one being used as transmitter and one as receiver (Olympus V303, transducer diameter: 12.7 mm, Panametrics Inc., Waltham, MA, USA), an arbitrary waveform generator (33250A, Agilent Technologies Inc., Santa Clara, CA, USA), an oscilloscope (DSO7054A, Agilent Technologies Inc., Santa Clara, CA, USA) and the simplified ankle model made of Perspex (Figure 4b). The ankle model was immersed in a water tank at room temperature. To hold the model tibia and model talus in their proper positions, as well as the US transducers, a custom-made Perspex frame was used (Figure 4b).

**Figure 5.**
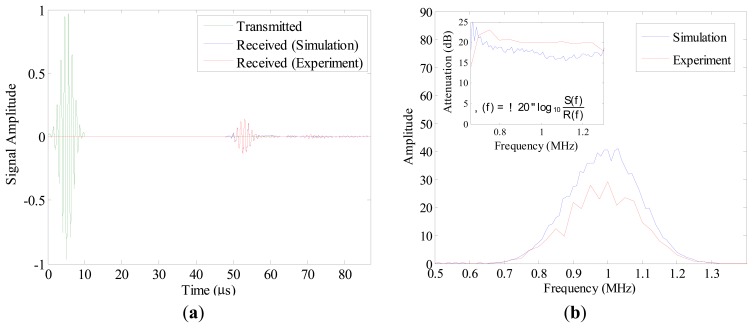
(**a**) Time function of the US transducer acting as transmitter, mean of 20 measurements and output of the reference simulation; (**b**) Amplitude spectra of the simulated signal and the mean signal recorded in experiment. In small window, attenuations [dB] in the simulated model and in the experiment are shown. Attenuations were calculated based on the provided formula, where α (f) is the attenuation [dB], log10 is the logarithm to the base 10, R(f) and S(f) respectively are the amplitude spectrum of the reference waveform (*i.e.*, input signal) and the amplitude spectrum of a receiver waveform (*i.e.*, received signal in experiment or in the reference simulation).

The transmitter was excited by a short normally distributed pulse with a peak-to-peak voltage of 9 V, a center frequency of 1 MHz, and a width of 4 μs. Before going through the measurements to validate the reference simulation results, the transmitter and the receiver were positioned facing each other in a straight line at a known distance. Having only water (*i.e.*, no joint model placed) in between the transmitter and the receiver, the signal was recorded and used as a time function for the source (Figure 5a) in the simulation models. Measurements performed for the validation were repeated 20 times, each time repositioning both US transducers.

### 2.3. Validation of the Reference Model

To evaluate the similarity between the results of the reference simulation and those of the experiment, the mean of the 20 measured output signals was calculated in the time domain and compared to the output signal of the simulation. The normalized maximum cross-correlation (NMCC) was used as a similarity measure:

(3) $$\text{NMCC}=\frac{\max\left|\left(f★g\right)\left[i\right]\right|}{\sqrt{\overset{n}{\underset{i=1}{\text{∑}}}f{\left[i\right]}^{2}}\cdot \sqrt{\overset{n}{\underset{i=1}{\text{∑}}}g{\left[i\right]}^{2}}}$$

where *f* and *g* are functions with a length of *n*, and (*f* ★*g*) [*i*] is the cross correlation of functions *f* and *g*. The reference simulation was assumed to represent reality sufficiently, if the calculated NMCC was within a margin of 5% (*i.e.*, NMCC ≥ 0.95).

### 2.4. Assessment of Parameters Effects

To assess effects of individual parameters on the output signals, results of each parametric simulation were compared to those of the reference simulation. For each case, NRMSE (Equation (2)) values were calculated and used for quantitative evaluation of changes in the reference output signal ensued as a result of variations in parameters. To discover whether variations in parameters mainly alter the shape of the reference output signal, the NMCC was determined as well, as it is invariant to time shift.

## 3. Results

The output signal of the reference simulation was compared to the mean of 20 measured output signals in the time (Figure 5a) and in the frequency domain (Figure 5b). The shapes of both output signals were highly similar, apart from a comparatively high flattening of the output signal of the reference simulation after 60 μs (Figure 5a). The similarity measure (*i.e.*, NMCC) between the output signals was found to be 0.97.

**Figure 6.**
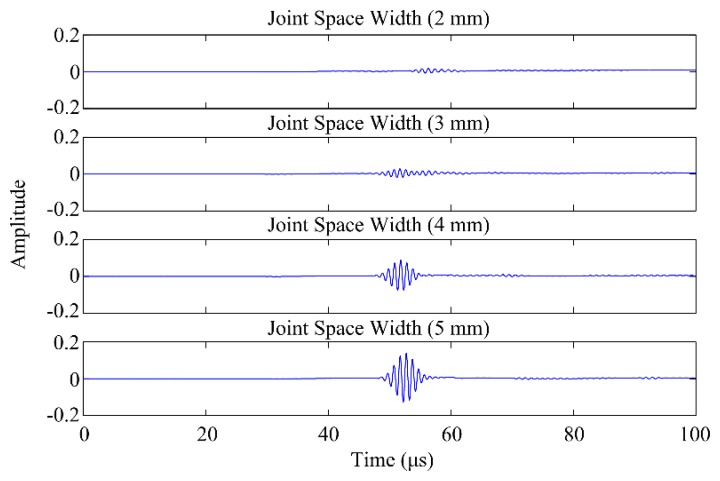
Outputs of the parametric models simulating the joint space width.

The amplitude of the output signal progressively increased when increasing the joint space (Figure 6). NRMSE and NMCC ranged from 6.29% (for joint space width: 4 mm) to 65.25% (for joint space width: 2 mm), and from 0.976 (for joint space width: 4 mm) to 0.391 (for joint space width: 2 mm), respectively (Table 2).

**Figure 7.**
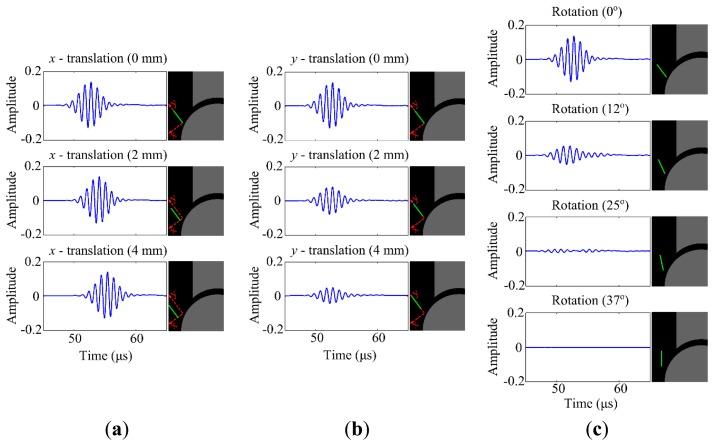
(**a**) Outputs of the parametric models simulating translation of US transducer (*i.e.* transmitter) in *x*-direction. Orientations of the transmitter and the receiver were kept identical to those in the reference model (*i.e.*, 37° from vertical); (**b**) Outputs of the parametric models simulating translation of US transducer (*i.e.*, transmitter) in *y*-direction. Orientations of the transmitter and the receiver were kept identical to those in the reference model (*i.e.*, 37° from vertical); (**c**) Outputs of the parametric models simulating rotation of US transducer (*i.e.*, transmitter). Position and orientation of the receiver were not changed and kept identical to those in the reference model.

No noticeable change in the amplitude of the output signal was present when translating the transducer in *x*-direction (Figure 7a), but, there was a clear phase shift. The amplitude of the output signal does considerably decrease when translating the transducer in *y*-direction (Figure 7b). The NRMSE measures support the finding that translation of the transducer in *x*-direction does not generate noticeable changes on the output signal in terms of amplitude, whereas translation in *y*-direction does (Table 2). The amplitude of the output signal is almost zero when the transducer is rotated 37° clockwise, which implies perpendicular alignment to the talar surface (Figure 7c). Analogous to the joint space width, variations in the transducer's rotational position cause high NRMSE (e.g., 78.02% for the transducer rotated 25° clockwise) and low NMCC (e.g., 0.632 for the transducer rotated 25° clockwise), supporting that apparent changes in the amplitude of the output signal occur.

**Figure 8.**
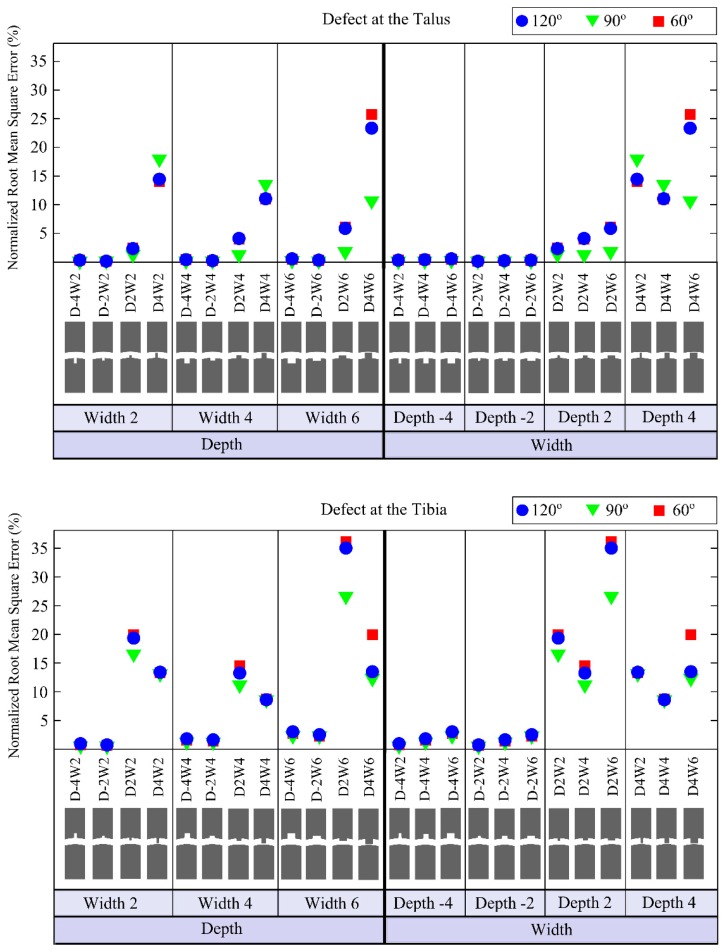
Normalized root mean square errors (NRMSE) for models simulating defects located on the talus (*i.e.*, first graph) and on the tibia (*i.e.*, second graph). The first three columns of the first and second graphs are provided to clearly represent the effects of varying defects depths when the defect width was fixed at 2 mm, 4 mm or 6 mm. The last four columns are presented to easily capture the changes related to the varying defects widths when the defect depth was fixed at −4 mm, −2 mm, 2 mm or 4 mm. Negative values for defect depth are used to represent actual holes in the bone, while positive values are used to describe positive defects, which may occur in the stage IV lesions (*i.e.*, detached and displaced osteochondral fragment). In the first and second graphs, depth and width of defect are represented by a “D” and a “W”, respectively. The minus sign “-” is used to describe negative defect, an actual hole in the bone (e.g., D-2W6 represents negative defect, *i.e.*, a hole, with a depth of 2 mm and a width of 6 mm).

**Figure 9.**
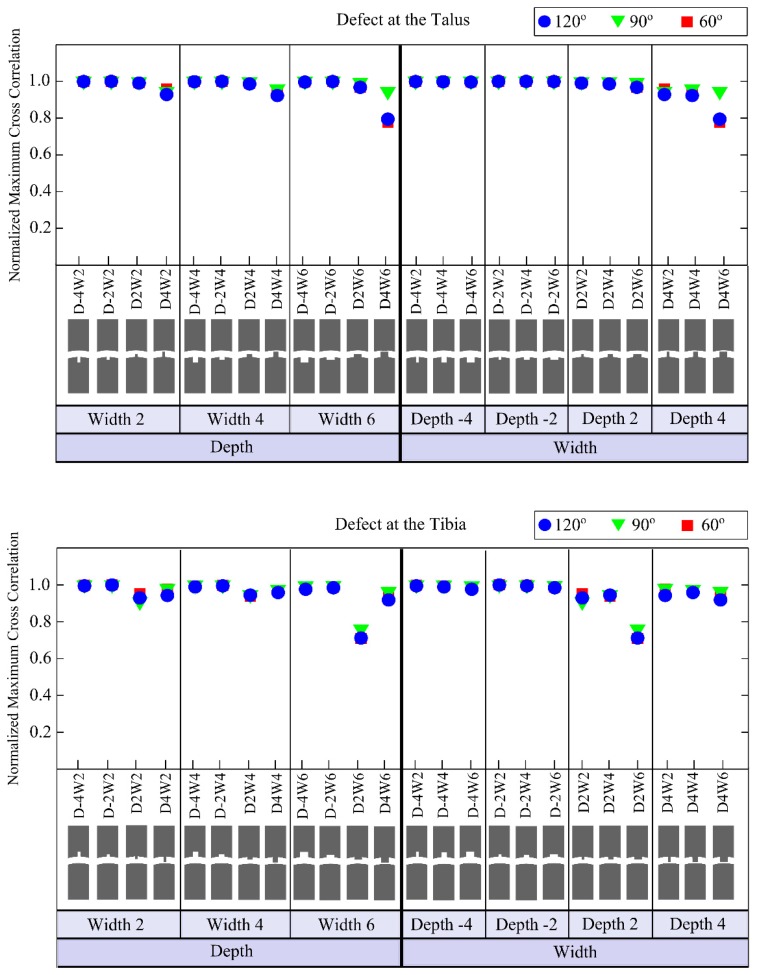
Normalized maximum cross correlation (NMCC) for models simulating defects located on the talus (*i.e.*, first graph) and on the tibia (*i.e.*, second graph). The first three columns of the first and second graphs are provided to clearly represent the effects of varying defects depths when the defect width was fixed at 2 mm, 4 mm or 6 mm. The last four columns are presented to easily capture the changes related to the varying defects widths when the defect depth was fixed at −4 mm, −2 mm, 2 mm or 4 mm. Negative values for defect depth are used to represent actual holes in the bone, while positive values are used to describe positive defects, which may occur in the stage IV lesions (*i.e.*, detached and displaced osteochondral fragment). In the first and second graphs, depth and width of defect are represented by a “D” and a “W”, respectively. The minus sign “-” is used to describe negative defect, an actual hole in the bone (e.g., D-2W6 represents negative defect, *i.e.*, a hole, with a depth of 2 mm and a width of 6 mm).

The defects having depths of −4 mm and −2 mm (*i.e.*, representing an actual hole in the bone) cause only small changes in the output signal (Figures 8 and 9) for any location on the tibia or the talus. The so-called positive defects with depths of 2 mm and 4 mm cause large differences in the output signal compared to the reference output as is clear from their higher NRMSE values (Figure 8).

The defect in the tibia with a depth of 2 mm and width of 6 mm shows the maximum deviation (*i.e.*, 36.16% NRMSE) from the reference signal (Figure 8). The maximum NRMSE for a talus defect (*i.e.*, 25.71%) is found for a depth of 4 mm and width of 6 mm (Figure 8).

## 4. Discussion

FDTD models of a joint space of the ankle were generated to determine the effects of variations of the joint space width, US transducer orientation and defect size and location on the propagation of a 1 MHz US pulse. The reference simulation results were validated against experimental measurements (Figure 5). From 60 μs, shape of output signal of the reference simulation differed slightly from that of the mean of 20 measured output signals (Figure 5a). Such a variation in shape may be results of reflections due to objects present in the actual setup, which were not included in simulations. However, considering the NMCC value of 0.97, which remains within a margin of 5%, it is admitted that the reference simulation agrees well with the experimental data.

A decline in the amplitude of the output signal was seen as a result of decreasing the joint space width (Figure 6). These findings indicate that to support the propagation of 1 MHz US pulse within the joint space of the ankle, it is recommended to distract the ankle joint. This is feasible for the majority of the population for up to 5 mm of joint space, as distraction is a standard procedure when performing ankle arthroscopy for treatment of OCD [18,19]. However, an increase in the joint space width does not mean that defects would relatively more easily be detected. Having a narrower joint space width might be more beneficial to detect defects, because the part of the signal ending up in any defect would be relatively larger in comparison to the part of the signal traveling through the joint space.

The NRMSE (*i.e.*, 3.96%–8064.2%) and NMCC (*i.e.*, 0.998–0.629) values of the models simulating the translation and rotation of the transducer showed no noticeable effect in *x*-direction, but a strong decrease of the output signal with translation in *y*-direction and rotation (Figure 7). Taking the high NMCC (*i.e.*, 0.998 and 0.996) and NRMSE (*i.e.*, 9.65% and 9.57%) for the models with transducer' translations in *x*-direction into account, one could conclude that the changes in the output signal mainly increased due to the shift of US pulse in time. By increasing the *y*-translation or rotation, a major part of US pulse will be sent through the bone into which the US beam has a limited ability to penetrate. This is not desired in the application of the proposed concept. From these simulations, it is concluded that the positioning of the transducers should be done with great care, and a device should be developed that can position the transducers with high reliability around the ankle joint of patients at multiple occasions, giving an optimal field of view for the transducers into the joint space.

**Figure 10.**
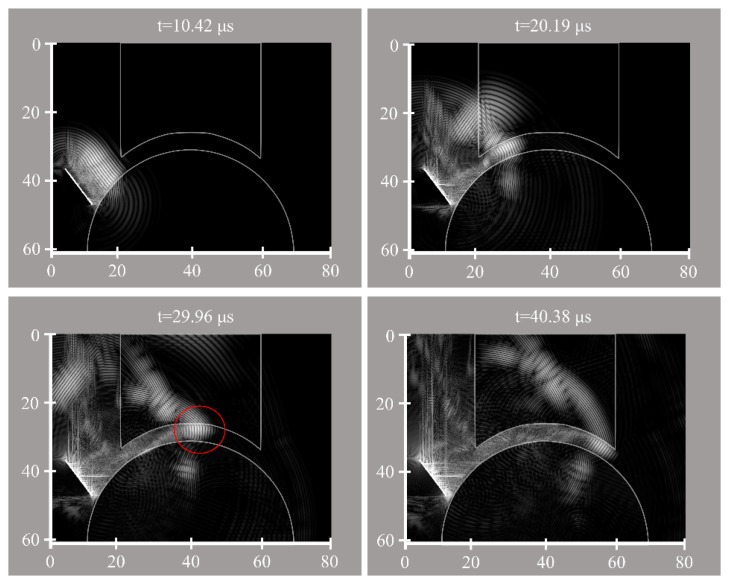
Simulated ultrasound pulse at t = 10.42 μs, 20.19 μs, 29.96 μs and 40.38 μs. The white bar is the transducer acting as transmitter. Red circle is used to emphasize US pulse guided along the tibia rim.

Analysing the results of variations in the defect size, it seems that positive defects, which may occur in the stage IV lesions (*i.e.*, detached and displaced osteochondral fragment) [23], made noticeable alterations to the output signal, whereas, negative defects (Figure 2) created relatively small changes (Figures 8 and 9). Increasing the width for negative talus defects did not seem to vary the output signal. However, the NRMSE values for increasing widths of the negative tibial defects did show what seems to be a linear trend (Figure 8) that might be helpful to determine tibial defect sizes with the proposed concept of wave propagation. No changes in the output signal due to variations in width of talus defects can be explained from Figure 10. The US pulse is guided along the tibia rim whereas it is less or not guided along the talus rim. Therefore, changes in the talus rim will not alter the wave propagation, but variations in the width of the tibia defects can be seen as a change in the amplitude of the signal (*i.e.*, the NRMSE values). This observation indicates that using the current configuration (e.g., frequency of the US transducers) detection of tibial defects would be easier than those of talus defects.

No distinguishable effect of the defect location (*i.e.*, defects located at 60°, 90° and 120°, Figure 2) was found on the output signal. Considering the fact that an accurate description of size and location of defect could help orthopedic surgeons in operative planning and in monitoring response to therapy [5], it would be more valuable if the location of defect besides its size could be determined using the proposed concept. In this respect, there is a need for further research on parameters (e.g., other frequencies), which might allow one to estimate the location of the defect.

One of the limitations of this study is the considerable simplification of the ankle joint. In the present model, cartilage and other soft tissues were not considered. As water may not be able to sufficiently replicate the absorption of US by soft tissues, the current results may somewhat differ from simulations including soft tissues in terms of US pulse amplitude. However, the general trends due to variations of the joint space width, and the translation and rotation of the US transducer are not expected to change. Furthermore, the alterations in the reference output signal due to defects are expected to be less noisy if the cartilage is also incorporated into the model, because the losses due to reflection and refraction at the water-cartilage interface would be smaller compared to the current water-Perspex interface, as water and cartilage have very similar densities.

Another limitation of this study is the omission of several parameters (e.g., the wave frequency, radius of the tibia and talus). To have a complete picture of US propagation within the joint space of the ankle, it is necessary to take also the omitted parameters into account and to evaluate their effects.

In this study, numerical modelling was limited to 2D to avoid relatively excessive computational cost of 3D models. Considering the other computational studies conducted to assess US interactions with cartilage [24], bone [25–27] and to evaluate US propagation within the joint space of a human knee [28], it is believed that 2D models are good enough for obtaining preliminary insight in the new concept.

In the present models, bony parts were represented by Perspex shapes so that simulations could be easily validated with experiments. A concern might be raised about use of a homogeneous joint mock-up to describe highly heterogeneous bony tissues. In the proposed concept, the focus is on the US wave sent through the ankle joint space and not through the bone itself, that is why both cortical bone and cancellous bone can be assumed homogeneous as suggested by White *et al.* [28]. Taking studies of White *et al.* [20,28,29] into account, the use of Perspex models therefore seems to be a reasonable starting point prior to the development of more complex models. In the future, simulations considering more realistic composition and structure of bony tissues will be developed and used to further investigate US wave travelling through the ankle joint and its use in detection of defects.

The current study has shed some lights on effects of variables (e.g., joint space width) on acoustic wave response and gives an idea on the feasibility of the new concept. Further research is required to determine acoustic parameters that can describe the entire joint space morphology and be robust enough to cope with individual variations. A next step will be the evaluation of the proposed concept using human ankle cadaver models as suggested by Tuijthof *et al.* [30]. For each human ankle cadaver model, size and location of defect will be estimated using US simulations and results will be compared with the real case (*i.e.*, the human ankle cadaver model).

The performance of the proposed diagnostic technique in identification of defects is expected to be better than that of the traditional US imaging modality. That is because the proposed technique has the potential to increase the percentage of the scanned articular surface to 100%, allowing US wave to propagate through the ankle joint. In comparison, only an estimated 50% of the talar surface could be visualized using traditional US imaging when the foot is in maximum plantar flexion [30]. Since only 80% of defects [5] are located in the area visualized by traditional US imaging, it cannot be used for diagnostic assessment. Moreover, the proposed diagnostic technique is more suitable for long-term follow of individuals having OCDs as compared to CT and MRI due to its non-invasiveness, compact set up and cost-effectiveness.

In summary, the current study provided a good start to investigate the feasibility of the concept to use acoustic waves traveling through the entire joint space for characterisation of changes in the joint shape due to the presence of OCDs. To the authors' best knowledge, this is an entirely new concept and the results indicate that it could be feasible provided that the effects of transducer orientation and joint space width are reduced. This requirement obliges development of a device that could fixate transducer orientation and joint space width in a reliable and repeatable manner.

## 5. Conclusions

The current study provided a good start to investigate the feasibility of the concept to use acoustic waves traveling through the entire joint space for characterisation of changes in the joint shape due to the presence of OCDs. To the authors' best knowledge, this is an entirely new concept and the results indicate that it could be feasible provided that the effects of transducer orientation and joint space width are reduced. This requirement obliges development of a device that could fixate transducer orientation and joint space width in a reliable and repeatable manner.

## Figures and Tables

**Table 1.** Material properties used in simulations.

	**Water**	**Plexiglas/Lucite**
ρ [kg/m^3^]	1000	1150
λ [MPa]	2241	5601
μ [MPa]	0	1392
ϕ [Pa·s]	9.998 × 10^−8^	0.01
η [Pa·s]	0.001	0.5
*C_L_* [m/s]	1497	2700
*C_S_* [m/s]	3.54491	1100.2
Longitudinal attenuation coefficient [dB/cm]	6.81479 × 10^−4^	5.12405 × 10^−2^
Shear attenuation coefficient [dB/cm]	153,953	0.559761

**Table 2.** Normalized root mean square error (NRMSE) and normalized maximum cross correlation (NMCC) for parametric models simulating the parameters related to the ankle joint (*i.e.*, joint space width) and to the US transducers (*i.e.*, translation and rotation of US transducer acting as transmitter). In each parametric simulation, only one parameter of the model was changed, and the other parameters were kept identical to those of the reference model.

**Variable**	**NRMSE (%)**	**NMCC**
Joint space width (2 mm)	65.25	0.391
Joint space width (3 mm)	29.75	0.643
Joint space width (4 mm)	6.29	0.976
Transducer *x*-translation (2 mm)	9.65	0.998
Transducer *x*-translation (4 mm)	9.57	0.996
Transducer *y*-translation (2 mm)	3.96	0.992
Transducer *y*-translation (4 mm)	11.72	0.929
Transducer rotation (37°)	8064.2	0.629
Transducer rotation (25°)	78.02	0.632
Transducer rotation (12°)	19.59	0.924
